# Uremic Myopathy and Mitochondrial Dysfunction in Kidney Disease

**DOI:** 10.3390/ijms232113515

**Published:** 2022-11-04

**Authors:** Eurico Serrano, Diana Whitaker-Menezes, Zhao Lin, Megan Roche, Maria Paula Martinez Cantarin

**Affiliations:** 1 Division of Nephrology, Department of Medicine, Sidney Kimmel Medical College, Thomas Jefferson University, 33 S 9th Street, Suite 700, Philadelphia, PA 19107, USA; 2 Sidney Kimmel Cancer Center, Thomas Jefferson University, Philadelphia, PA 19107, USA

**Keywords:** mitochondria bioenergetics, uremic myopathy, glycolysis, fatty acid oxidation, OXPHOS

## Abstract

Alterations in muscle structure and function in chronic kidney disease (CKD) patients are associated with poor outcomes. As key organelles in muscle cell homeostasis, mitochondrial metabolism has been studied in the context of muscle dysfunction in CKD. We conducted a study to determine the contribution of oxidative metabolism, glycolysis and fatty acid oxidation to the muscle metabolism in CKD. Mice developed CKD by exposure to adenine in the diet. Muscle of CKD mice showed significant weight loss compared to non-CKD mice, but only extensor digitorum longus (EDL) muscle showed a decreased number of fibers. There was no difference in the proportion of the various muscle fibers in CKD and non-CKD mice. Muscle of CKD mice had decreased expression of proteins associated with oxidative phosphorylation but increased expression of enzymes and transporters associated with glycolysis. In cell culture, myotubes exposed to uremic serum demonstrated decreased oxygen consumption rates (OCR) when glucose was used as substrate, conserved OCR when fatty acids were used and increased lactate production. In conclusion, mice with adenine-induced CKD developed sarcopenia and with increased glycolytic metabolism but without gross changes in fiber structure. In vitro models of uremic myopathy suggest fatty acid utilization is preserved compared to decreased glucose utilization.

## 1. Introduction

Uremic myopathy is the term used to refer to changes in muscle structure and function that are present in patients with advanced kidney disease, including sarcopenia, cachexia, protein energy wasting and muscle atrophy [1,2]. The clinical presentation of uremic myopathy includes cardiomyopathy, muscle wasting, weakness, low endurance and fatigue [3,4]. Muscle wasting can lead to insulin resistance, accelerated cardiovascular disease, longer hospital stays and increased mortality in patients with kidney disease [5,6,7].

Muscle loss or atrophy is a process widely observed with advanced age, but it is also prevalent in patients with chronic diseases including cancer, heart failure and chronic kidney disease (CKD) [4,7]. Muscle loss is more prevalent in patients with CKD than the non-CKD population and prevalence increases as kidney disease progresses to end-stage renal disease (ESRD) [8]. Muscle loss and decreased muscle function in patients with CKD also contributes to the clinical syndrome of frailty. Around 7% of the elderly population over 65 meet the definition of frailty while frailty affects up to 70% of ESRD patients over age 65 years old and affects 47% of younger adults (18–64 years old) on dialysis [9,10,11,12].

Multiple mechanisms may contribute to uremic myopathy in kidney disease patients [13,14], including inflammation, exposure to elevated levels of reactive oxygen species (ROS) [15], malnutrition and metabolic acidosis [16,17]. Mitochondria play a central role in muscle cell metabolism, energy supply, regulation of energy-sensitive signaling pathways, reactive oxygen species (ROS) production, calcium homeostasis and the regulation of apoptosis [18]. As key organelles in muscle cell homeostasis, mitochondrial metabolism has been studied in different disease processes including aging, muscular dystrophies and even insulin resistance. Muscle of patients with kidney disease has lower mitochondrial content and altered morphology with increased fragmentation, and swollen cristae [19,20]. Additionally, mitochondrial dynamics are altered in CKD with increased fission [20]. The process of mitochondrial renovation or mitochondrial biogenesis is also a central component of skeletal muscle function and structure. CKD patients not only have decreased mitochondrial content, with a lower mitochondrial DNA (mtDNA) copy number, but decreased mitochondrial biogenesis and impaired overall function [19,21]. Impaired mitochondrial metabolism, despite preserved oxygen supply [22,23,24,25,26], is a key process leading to uremic sarcopenia, although the mechanisms that lead to mitochondrial dysfunction in CKD remain elusive.

The aim of the study is to determine the contribution of oxidative metabolism, glycolysis and fatty acid oxidation to the muscle metabolism in chronic kidney disease using in vivo and in vitro models of advanced kidney disease.

## 2. Results

### 2.1. Characterization of Mouse Kidney Disease Model by Using Adenine Supplementation in Diet

**Figure 1:**
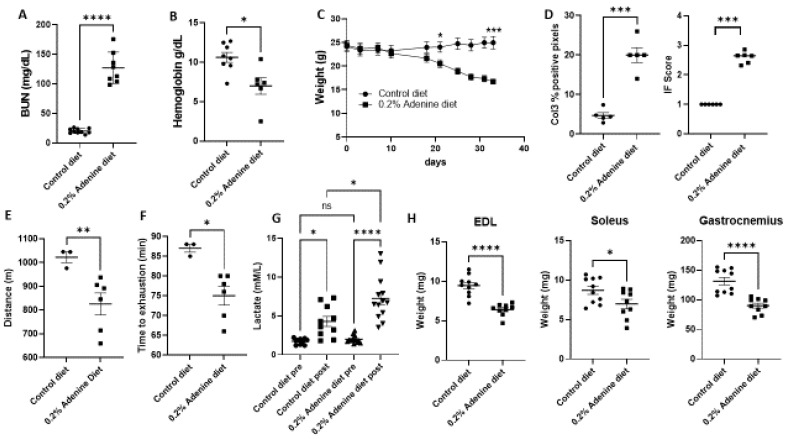
Phenotype of mice exposed to a 0.2% adenine diet. Male and female C57BL/6 mice 8 to 10 weeks old were fed a standard diet or 0.2% adenine-containing diet ad libitum for 4 weeks (**A**) BUN level (mg/dL) in adenine diet fed mice versus control mice. (**B**) Differences in hemoglobin (g/dL) between control and adenine diet group. (**C**) Differences in weight during adenine and control diet supplementation. (**D**) Collagen 3 immunostaining and interstitial fibrosis (IF) score from Trichrome stain from renal tissue of animals treated with control or adenine diet for 4 weeks. Intensity of collagen 3 was measured using Aperio Imagescope and analyzed by positive pixel count v9 algorithm. (**E**,**F**) Results of treadmill test comparing muscle endurance in control and adenine treated mice. (**E**) Distance run in meters. (**F**) Time to exhaustion in minutes. (**G**) Blood lactate levels pre- and post- treadmill test. (**H**) Muscle weights from extensor digitorum longus (EDL), Soleus and gastrocnemius from control mice and mice exposed to adenine diet. Unpaired t test with Welch’s correction, *n* = 8–12 per test, error bars represent SEM * *p* ≤ 0.05, ** *p* < 0.01, *** *p* < 0.001, **** *p* < 0.0001. BUN, blood urea nitrogen. IF, interstitial fibrosis, Col3, Collagen 3.

To study the contribution of mitochondrial metabolism to muscle dysfunction in kidney disease, we used a murine model of kidney disease by supplementing adenine in the diet. Kidney disease was demonstrated by a significant increase in blood urea nitrogen (BUN) in the CKD groups (Figure 1A). Mice fed an adenine diet also showed other characteristics of advanced kidney disease, including anemia (Figure 1B) and marked weight loss (Figure 1C). Adenine exposure induced changes in kidney histology including interstitial fibrosis measured by collagen 3 accumulation in renal cortex and Trichrome stain (Figure 1D, Supplementary Figure S1) as well as presence of tubular atrophy and 2,8-dihydroxyadenine crystals. Mice exposed to an adenine diet showed decreased exercise endurance in a forced endurance treadmill test (Figure 1E,F). Mice exposed to adenine ran shorter distances and reached exhaustion earlier than mice exposed to a normal diet. The treadmill protocol gradually increased the speed over time to a maximum of 26 m/min, when oxygen consumption in C57BL/6 mice reaches approximately 80% of VO_2_ max, which is considered high-intensity exercise [27]. At high intensity, most of the energy utilized by muscle comes from glycolytic metabolism and results in lactate release from muscle fibers and accumulation in plasma [26]. Consequently, mice exposed to adenine showed an increase in blood lactate levels post-exercise that was significantly higher than the lactate levels observed after exercise in control mice (Figure 1G). The higher post-exercise lactate in mice exposed to adenine suggest a prominent glycolytic response in uremic muscle. To determine if the functional muscle changes are coupled with muscle structural changes, we weighted the Soleus, extensor digitorum longus (EDL) and gastrocnemius muscles of mice exposed to adenine and compared to muscle from mice on a control diet. Adenine exposed mice showed lower muscle weights than control mice in the three muscle groups (Figure 1H). In sum, mice exposed to an adenine-based diet developed CKD with high BUN levels, anemia, weight loss and decreased exercise endurance. Furthermore, mice with CKD had higher lactate levels post-exercise than control mice with loss of muscle mass, reflecting a prominent glycolytic metabolism.

### 2.2. Muscle Fiber Typing Profile of Mice Exposed to Adenine

**Figure 2:**
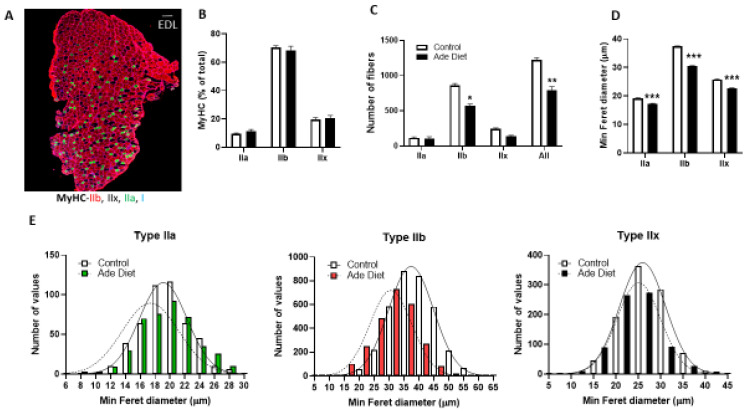
Extensor digitorum longus (EDL) muscle fibers characteristics in mice with kidney disease. Mice were exposed to a 0.2% adenine-containing diet to induce kidney disease. Muscle fiber composition and structure were compared between mice that were exposed to adenine versus control diet. (**A**) Representative photograph of EDL muscle myosin heavy change (MyHC) staining in control mice (Scale bar = 100 microns). (**B**) Relative fiber-type composition (**C**) Number of fibers. (**D**) Minimum Feret diameter. (**E**) Frequency histograms of fiber size of EDL muscles measured as minimum Feret diameter (n = 4 in each group measuring all the fibers in each section). Unpaired t test with Welch’s correction (Type IIA fibers) and Mann–Whitney test (Type IIB and IIX), error bars represent SEM * *p* ≤ 0.05, ** *p* < 0.01, *** *p* < 0.001.

**Figure 3:**
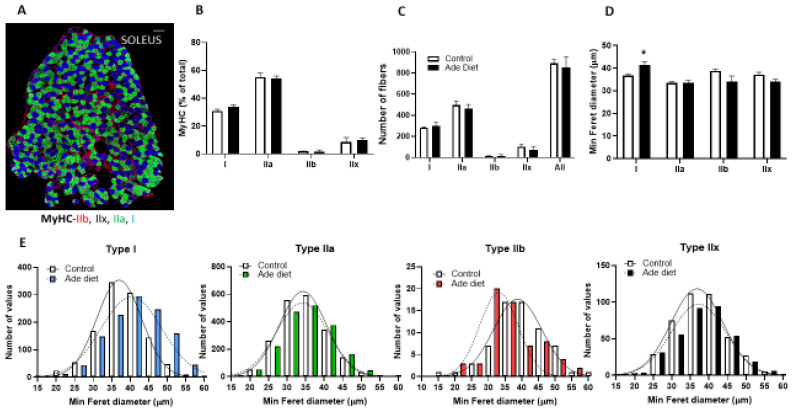
Soleus muscle fiber characteristics in mice with kidney disease. Mice were exposed to a 0.2% adenine-containing diet to induce kidney disease. Muscle fiber composition and structure were compared between mice that were exposed to adenine versus control diet. (**A**) Representative photograph of Soleus muscle MyHC staining in control mice (Scale bar = 100 microns). (**B**) Relative fiber-type composition. (**C**) Number of fibers. (**D**) Minimum Feret diameter of Soleus fibers. (**E**) Frequency histograms of fiber size of Soleus muscles measured as minimum Feret diameter (n = 4 in each group measuring all the fibers in each section). Unpaired t test with Welch’s correction (Type IIB, IIX fibers) and Mann–Whitney test (Type I and IIA), error bars represent SEM * *p* ≤ 0.05.

To determine if the decreased exercise endurance in CKD mice is due to changes in muscle fiber composition, we stained sections of EDL and Soleus muscle from adenine exposed and control mice with antibodies specific for the different isoforms of myosin heavy chain (MyHC). We studied EDL muscle as an example of fast twitch glycolytic muscle and Soleus muscle as an example of slow oxidative muscle. The fiber type profile of CKD mice was similar to controls, with the fast EDL predominantly composed of type IIb and IIx fibers, with few type IIa fibers and the slow Soleus predominantly composed of type I and IIa fibers, with few type IIx fibers (Figure 2A and Figure 3A). Figure 2 shows the results in EDL muscle. Mice exposed to adenine diet demonstrate similar fiber composition than control mice with similar percentage of type IIa, IIb and IIx fibers (Figure 2B). The number of IIb fibers and overall number of fibers was lower in mice exposed to adenine (Figure 2C). We used minimum Feret diameter as a measure of the fiber size and we demonstrated that in mice exposed to adenine, IIa, IIb and IIx fibers were smaller than in control mice (Figure 2D,E). In summary, EDL muscle of mice that develop CKD had fewer and smaller fibers than control mice, although we did not appreciate a change in the proportions of different fiber types. Figure 3 shows data from Soleus muscle. Similar to the results described for the EDL muscle, mice exposed to adenine diet demonstrate comparable fiber type composition to control mice, and specifically, there was a similar percentage of type I, IIa, IIb and IIx fibers (Figure 3B). The number of fibers in every subtype and overall number of fibers was not different in mice exposed to adenine (Figure 3C). In contrast, type I fibers had bigger Feret diameter in mice exposed to adenine than in control mice (Figure 3D,E). In summary, Soleus muscle of mice that develop CKD had a similar number of fibers, a higher proportion of the different fiber types and bigger type I fibers than control mice.

### 2.3. Muscle of Mice with CKD Have Decreased Expression of OXPHOS Proteins and Increased Expression of Glycolytic Proteins

**Figure 4:**
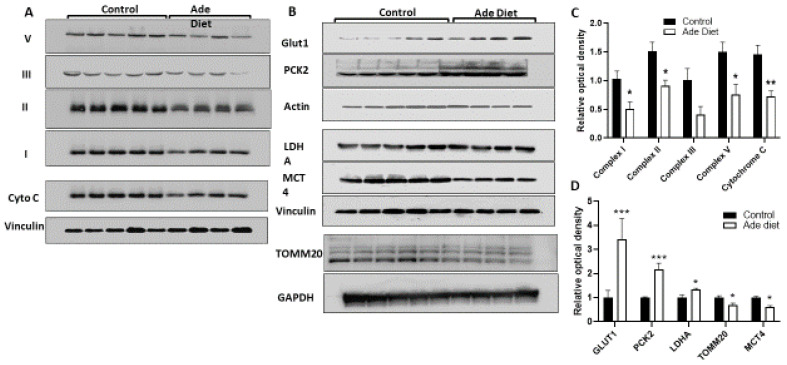
OXPHOS profile of gastrocnemius muscle from CKD mice compared to controls. (**A**) OXPHOS profile and cytochrome C were evaluated by Western blot analysis on gastrocnemius muscle of mice with CKD and controls. The OXPHOS panel includes antibodies against the subunits of complexes (I–V) that are labile when the respective complex is improperly assembled. (**B**) Western blot of several mitochondrial enzymes involved in glycolysis. (**C**) OXPHOS panel protein expression normalized by densitometry analysis. (**D**). Mitochondrial protein expression normalized by densitometry analysis. (n = 4–5). Vinculin, Actin or GAPDH were used as an equal loading control. Unpaired t test with Welch’s correction, error bars represent SEM. * *p* ≤ 0.05, ** *p* < 0.01, *** *p* < 0.001. GLUT1: Glucose transporter 1, PCK2: Phosphoenolpyruvate Carboxykinase 2, Mitochondrial, LDHA: Lactate Dehydrogenase A, MCT4: Monocarboxylate Transporter 4, TOMM20: Translocase of the outer mitochondrial membrane complex subunit 20.

To gain insight into the mechanisms by which kidney disease alters muscle function, we studied muscle mitochondrial metabolism in mice with kidney disease. Mitochondrial oxidative phosphorylation (OXPHOS) is the most efficient mechanism to produce ATP and the main pathway used for muscle bioenergetics in aerobic conditions. Mitochondrial OXPHOS can utilize glucose or fatty acids as substrates for ATP production. In anaerobic conditions, glycolysis with lactate production is a quick but energetically inefficient way to produce energy or ATP. We studied the OXPHOS profile and cytochrome C, an essential component of the respiratory electron transport chain (ETC) that transfers electrons from complex III to complex IV, in mice with kidney disease and compared to control mice by Western blot. The OXPHOS panel includes antibodies against the subunits of complexes (I–V) that are labile when the respective complex is improperly assembled. Thus, changes in expression levels of these subunits reflect alterations in the assembly of the respective complex and by extension, decreased function. Mice with kidney disease have decreased expression of complex I, II, III and V as well as cytochrome C (Figure 4A,B), suggesting that those complexes as well as Cytochrome C are dysfunctional. We then studied additional enzymes and transporters that regulate glycolysis and OXPHOS. Concordant with our data from the labile subunits of the mitochondrial complexes, transporter of the outer mitochondrial membrane subunit 20 (TOMM20) expression was decreased in muscle of mice with CKD compared to control. We also studied several enzymes associated with the glycolytic process, including glucose transporter 1 (GLUT1) and monocarboxylate transporter 4 (MCT4), the main lactate exporter from cells, both rate limiting proteins of glycolytic flux [28], lactate dehydrogenase (LDHA), the enzyme than coverts pyruvate to lactate, and phosphoenolpyruvate carboxykinase 2 (PCK2), the cataplerotic enzyme that links the Tricarboxilic acid cycle (TCA) cycle with glycolysis. In contrast to the OXPHOS data, LDHA, GLUT1, PCK2 and MCT4 had increased expression in muscle of CKD mice compared to control mice (Figure 4C,D). In summary, muscle of mice with CKD have decreased expression of OXPHOS related proteins but with increased expression of enzymes and transporters associated with glycolytic metabolism.

### 2.4. Cells Exposed to Uremic Serum Have Prominent Glycolytic Metabolism with Decreased Glucose-Derived OXPHOS and Preserved Fatty Acid Oxidation

**Figure 5:**
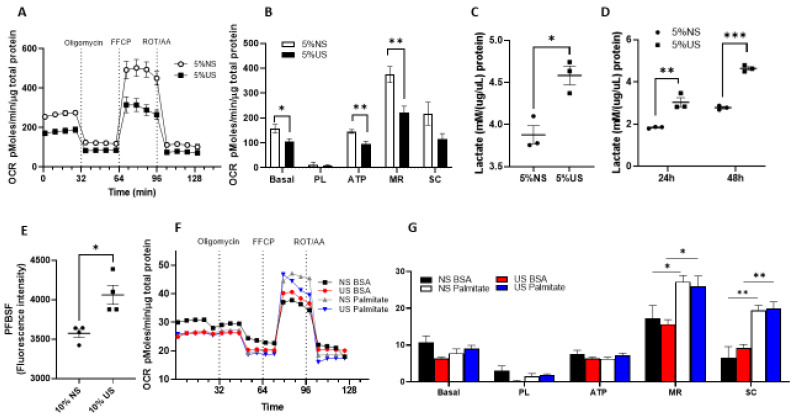
Cells exposed to uremic serum have prominent glycolytic metabolism with decreased OXPHOS. (**A**) Representative time course data of oxygen consumption rates (OCR) measurements in C2C12 differentiated myotubes treated with 5% control and uremic serum for 24 h. (**B**) OCR-aggregated data. (**C**) Lactate production in differentiated myotubes exposed to 5% normal and uremic serum after 24 h of serum treatment. (**D**) Lactate production in differentiated myotubes exposed to normal and uremic serum for 24 h. After 24 h, the serum is washed out and lactate production is measured 24 h and 48 h later in control media. (**E**) Extracellular H_2_O_2_ production in cells treated with uremic and control serum. (**F**) Representative time course data for OCR from fatty acid oxidation (FAO) in myotubes treated with 5% normal or uremic serum. (**G**) FAO aggregated data. Data from OCR experiments represent the mean of six technical replicates from two representative experiments. Data from lactate experiment represent the mean of four technical replicates from two representative experiments. Unpaired t test with Welch’s correction, error bars represent SEM. * *p* ≤ 0.05, ** *p* < 0.01, *** *p* < 0.001. OCR, oxygen consumption rates. PFBSF, pentafluorobenzenesulfonyl fluorescein. PL, proton leak. ATP, ATP production. MR, maximal respiration. SC, spare respiratory capacity. NS, normal serum. US, uremic serum.

To determine the contribution of glucose-derived OXPHOS, fatty acid oxidation (FAO) and glycolysis to muscle metabolism in kidney disease, we used the C2C12 myoblast cell line after exposure to different concentrations of uremic and normal human serum as we previously described [29]. Cells exposed to uremic serum had lower rates of oxygen consumption analyzed by SeaHorse with decrease in basal oxygen consumption, ATP production, as well as maximal respiratory capacity (Figure 5A,B). To analyze glycolysis rates, we studied lactate production in cells exposed to uremic serum versus normal serum. As depicted in Figure 5C, lactate production increases in cells exposed to uremic serum compared to cells exposed to normal serum. Furthermore, even after removing the serum exposure, cells previously in contact with uremic serum continued to generate higher lactate at 24 h and 48 h (Figure 5D). Lactate data in C2C12 myotubes were replicated in undifferentiated myoblast and differentiated myotubes exposed to 10% normal and uremic serum (Supplementary Figure S2A–C). Reactive oxygen species and its most abundant species, H_2_O_2_, are generated with defective OXPHOS, and we observed higher levels of extracellular H_2_O_2_ in cells exposed to uremic serum compared to controls measured by Pentafluorobenzenesulfonyl fluorescence (PFBSF) (Figure 5E). We also studied FAO in cells exposed to uremic serum compared to normal serum, and as shown in Figure 5F, FAO rates are similar with uremic serum exposure including similar responses to palmitate for maximal respiration and spare respiratory capacity (Figure 5G). In summary, muscle cells exposed to uremic serum utilize fatty acids more efficiently than glucose as a source of energy production.

## 3. Discussion

Our study demonstrates that mice with advanced kidney disease have altered muscle structure and function consistent with the phenotype of uremic myopathy. Mice with CKD suffer significant muscle loss, decreased assembly of electron transport chain proteins as well as enhanced glycolytic metabolism. Despite muscle loss, glycolytic and oxidative muscle groups maintain the proportions of the different fiber types in our in vivo model of adenine-induced nephropathy. In vitro models of advanced kidney disease, muscle cells reveal that exposure to uremic serum impairs oxygen-derived OXPHOS but preserves FAO.

Loss of muscle mass with CKD has been previously documented in patients as well as in animal models [25,30]. Muscle tissue mass decreases because of decreased size (atrophy) and number (hypoplasia) of muscle fibers [4]. Mice exposed to adenine in our study also suffer from muscle loss, depicted by decreased muscle weights in the CKD group. Interestingly, when we studied EDL and Soleus muscle for fiber composition, we only found reduced number of fibers and decreased diameter in the glycolytic EDL muscle. Possible explanations for the decreased weight with conserved fiber number and size could be an increased myofibrillar adipose tissue content or edema. Human studies have shown that intramuscular triglyceride accumulation with insulin resistance is a common feature in CKD and that the lipid deposition affects predominantly Type I fibers [31]. Therefore, it is possible that the decrease in muscle weight with preserved number of fibers and diameter in the Soleus muscle of our adenine-exposed mice is due to increased lipid deposition.

Prior studies in murine models have shown a muscle fiber switch from oxidative to glycolytic fibers that could explain the lack of exercise endurance and early fatigability in CKD mice [25,32]. Muscle fibers have been historically classified as fast-twitch fibers with predominantly glycolytic metabolism or slow-twitch fibers with a preponderant oxidative metabolism. Myosin ATPase staining correlates with speed of muscle shortening and has led to classifying fibers as type II (fast twitching) or type I (slow twitching) fibers. Based on fiber’s succinate dehydrogenase (SDH) staining to reflect oxidative metabolism, fibers are subclassified as fatigue resistant (FR with increased SDH activity) or fast fatigable (FF). Fibers can be classified using both ATPase and SDH staining as type IIa (FR or fast oxidative-glycolytic) fibers or IIb (FF or fast glycolytic) fibers. Our study utilized monoclonal antibodies against myosin heavy chain (MyHC) isoforms to be able to differentiate between fiber types and this facilitated the subclassification of type IIx fibers (with twitch properties similar to IIa and IIb fibers and resistance to fatigue between IIa and IIb fibers). Fiber typing characterization in murine and human studies have not shown congruent results [25,32]. Specifically, Tamaki et al. showed a decrease in the proportion of type I (slow oxidative) and IIa (fast oxidative glycolytic) and a relative increase in IIB (fast glycolytic) muscle fibers in mice with CKD by SDH staining [25]. On the other hand, Higashihara et al. demonstrated loss of fast type II fibers predominantly in his murine model of CKD by myosin antibody staining in gastrocnemius muscle [32].

These discrepancies are not only seen in murine studies but also in human studies. In a recent cohort of fit subjects on maintenance dialysis awaiting kidney transplantation, skeletal muscle studies demonstrated preserved muscle fiber cross-sectional area, and no increase in sarcopenia, despite lower endurance capacity compared to controls. Fiber typing studies of vastus lateralis muscle in the same cohort demonstrated a lower percentage of MyHC type I fibers with higher percentage of other MyHC fibers, reflecting a switch from slow to fast fiber type [33]. On the other hand, in a similar study by Lewis et al. [34], on vastus lateralis muscle biopsies of subjects on maintenance dialysis, there was a small but significant increase in type I fibers with a small decrease in IIx fibers. Although the results of the two studies may seem contradictory, in terms of the frequency of type I fibers in CKD, there were methodological differences. Lewis et al. also studied SDH activity within individual fibers of vastus lateralis and demonstrated lower SDH activity in each fiber subtype in dialysis patients, consistent with the concept of decreased oxidative capacity in muscle of CKD patients independently of the fiber type.

Our results in mice differ slightly from data with other models and highlight the complexity of these muscle-profiling studies. The most commonly murine models used in the literature are 5/6 nephrectomy and adenine dietary supplementation which affect GFR differently and may induce different degrees of muscle dysfunction. Furthermore, the adenine model is associated with decreased oral intake due to the aversion to adenine in mice despite the use of casein to mask adenine taste, which may result in more significant muscle loss and dysfunction. Human muscle is not as compartmentalized as that in mice, with a mix of both glycolytic and oxidative fibers in muscle groups without a clear preponderant type in human muscle. MyHC staining reflects gross metabolic groups but specific metabolic stains such as SDH for oxidative potential or α-Glycerophosphate Dehydrogenase for glycolytic potential may be needed to better delineate metabolic capacity within specific groups.

We studied the mitochondrial electron transport chain (ETC) supercomplexes in our model of CKD as a surrogate for muscle OXPHOS capacity. Dysfunction of ETC complexes has been described in age-related loss of kidney function [35] and in AKI [36]. Gastrocnemius muscle of mice with CKD demonstrated decreased mitochondrial ETC supercomplexes and cytochrome C compared to control mice corroborating our prior data in C2C12 cells exposed to uremic serum [29]. Correct assembly of the complex subunits is necessary for proper function and activity of ETC, which is critical for mitochondrial fitness. Our data on expression of metabolic enzymes also support a preponderant glycolytic metabolism with increased expression of GLUT1, LDHA and PCK2. In contrast, Thome et al. [26] described intact protein content of ETC subunits but decreased activity of the mitochondrial dehydrogenase enzymes pyruvate dehydrogenase and alpha ketoglutarate dehydrogenase. Functionally, the authors propose that a defect in matrix dehydrogenase activity is the main driver of decreased mitochondrial metabolic activity. Impaired mitochondrial complex formation despite conserved absolute complex protein values has been described in muscle of diabetic patients [37], so it is possible that the same is true in muscle of mice with CKD. To better delineate this discrepancy and since dehydrogenases plays a key role in oxidation of glucose, we studied uremic muscle utilization of fatty acids. Our in vitro data using a cell model of uremic muscle demonstrated that there is impaired mitochondrial metabolism when glucose is used as the main energy substrate, but intact oxygen consumption rates are demonstrated when fatty acids are utilized. We also showed a preponderance of glycolytic metabolism and ROS production demonstrated by the production of lactate and H_2_O_2_ when muscle cells are exposed to uremic serum. Reductions in muscle OXPHOS in the context of CKD has been demonstrated by different groups including by our lab [25,38]. We previously showed that there is decreased TOMM20 expression and mitotracker activity in C2C12 cells exposed to uremic serum as well as ESRD patients [29]. Thome et al. demonstrated decreased oxygen consumption rates in isolated mitochondria from mice with CKD using several carbohydrates as substrates. In contrast to our data, the authors also demonstrated a decrease in OXPHOS conductance using fatty acids [26]. These different results could be explained by the difference in the in vitro models. Studies on isolated mitochondria have shown that mitochondrial changes in structure and function secondary to different disease processes are parallel but augmented when compared to studies on intact mitochondria in permeabilized fibers [39,40]. Thus, it is possible that changes in FAO with uremia have been exacerbated as they were studied in isolated mitochondria. Studies that have focused on the role of the different metabolic pathways in uremic sarcopenia are lacking. Conversely, the contribution of FAO, glycolysis and glucose-derived oxidative phosphorylation is better delineated in kidney tubular epithelial cells in CKD. Proximal tubular cells have high baseline energy demands and have high number of mitochondria. It is widely recognized that cells with high baseline metabolic rates use FAO as their main metabolic pathway due to the higher efficiency of fatty acid oxidation in comparison with oxidation of glucose. Kidney tubular epithelial cells in CKD have defects in FAO [41,42] and increased glycolysis [43,44,45]. Since mitochondrial FAO contributes a large proportion to the body’s energy needs in fasting and in situations of metabolic stress, it is possible that uremic muscle, which has reduced dehydrogenase activity including reduced PDH activity, will switch to FAO from glucose catabolism as a critical source of energy support. Studies in permeabilized myofibers may help to better delineate the contribution of different substrates to muscle energy production in CKD. ROS contributes to increased catabolism, increases muscle atrophy and affects muscle contractility [46,47]. Patients with CKD have higher peripheral markers of oxidative stress [48,49] and there is also increased ROS production in uremic muscle, as we demonstrated in this study. The contribution of locally produced ROS versus systemic ROS in CKD patients to the progression of uremic myopathy is an area that has not been extensively studied and deserves further attention in future studies [15].

Our study has several limitations. The model of adenine-induced nephropathy leads to significant weight loss due to the limited food intake as adenine is poorly tolerated. Hence, this model of CKD has a component of cachexia that may not be widely represented in the human disease. The young age of the mice commonly used for the CKD models (8 to 15 weeks old) could influence muscle physiology as the muscle development is still immature. The adenine model induces tubule-interstitial kidney disease. Other kidney diseases, such as diabetic kidney disease, have intrinsic metabolic changes that can also affect muscle metabolism and may have different effects on muscle performance since diabetes mellitus, independently of CKD, can affect muscle function.

In summary, our model of tubulo-interstitial CKD has sarcopenia and decreased energy endurance with increased oxidative stress and reduced glucose-derived OXPHOS, but without gross fiber typing changes in oxidative and glycolytic muscle groups with preserved FAO. Detailed studies of energy utilization with different substrates in muscle of advanced CKD models and patients are needed in the future to focus on specific therapeutic approaches with focus in supporting specific metabolic pathways.

## 4. Materials and Methods

Animal care and use. Male and female C57BL/6 mice 8 to 10 weeks old (Strain 000664) were obtained from Jackson Laboratories and fed a standard diet (PicoLab^®^ Rodent Diet 20, 5053) or 0.2% adenine-containing diet (Modified LabDiet^®^ PicoLab^®^ Rodent Diet 20, 5053, with 0.2% adenine A8626; Sigma) ad libitum for 4 weeks. All animal procedures were in compliance with protocols approved by the Institutional Animal Care and Use Committee at Thomas Jefferson University. All methods were carried out in accordance with relevant NIH guidelines and regulations.

### 4.1. Exercise Tolerance Test

The exercise tolerance test was assessed using a motorized Exer3/6 Treadmill system with shock counter (Columbus Instruments). Mice were acclimated for 3 consecutive days to the treadmill, 2 days before the test with an experimental run for 10 min at 0 incline. The test was performed with 0 incline and started at 0 m/min to 5 m/min (20 s of speed transition) maintained for 12 min with progressive speed increases for a total running time of 90 min. Briefly, speed increased from 2.5 m/min (1 min of transition for each speed increase) until 10 m/min every 12 min. For the next phase, the treadmill speed was increased 2.5 m/min every 10 min until it reached a speed of 20 m/min. For the final phase, the speed was increased every 6 min until the treadmill reached a final speed of 25 m/min that was maintained for 4 min. Blood lactate levels were measured pre- and post-experimental run using a Lactate Plus Meter (Nova Biomedical, Waltham, MA, USA).

### 4.2. Blood Urea Nitrogen (BUN)

Analyzed from murine blood obtained by intracardiac puncture using the QuantiChrom^TM^ Urea Assay Kit (DIUR-100) as directed by the manufacturer.

### 4.3. Complete Blood Count (CBC)

Analyzed in 50 µL of murine whole blood in a GENESISTM Veterinary Hematology System by the Translational Research/Pathology Core at the Sidney Kimmel Cancer Center.

### 4.4. Trichrome Stain and Quantification

Wild type kidneys from mice on adenine diet were fixed in formalin, paraffin-embedded and four micron sections were cut and stained with Trichrome stain at the SKCC Translational Research/Pathology Shared Resource facility at Thomas Jefferson University. The amount of fibrosis in each kidney sample (n = 6) was scored by two independent observers at 20× magnification using the scale 1 = <25%, 2 = 25–50%, 3 = 50–75%, 4 = >75%. A total of 21–25 cortical fields were scored and the average overall score was generated for each kidney [50,51,52,53].

### 4.5. Collagen III Immunohistochemistry and Quantification

Four micron paraffin sections of mouse kidney were stained with goat antibody to collagen III alpha 1/COL3A1 (Novus Biologicals #NBP1-26547) using a three-step avidin-biotin horseradish peroxidase method (Vector Labs, Newark, CA, USA). Briefly, sections were deparaffinized, rehydrated and antigen retrieval was performed using 10 mM sodium citrate, pH 6.0 buffer for 10 min using an electric pressure cooker. After cooling, sections were blocked with 3% hydrogen peroxide and incubated overnight at 4 °C with 10% normal rabbit serum. Primary antibody was incubated for one hour at room temperature, followed by blocking for endogenous biotin using the Biocare Medical Avidin Biotin kit (#AB972H). Secondary and tertiary steps were for 30 min each and labeling for collagen 3 was detected using the SignalStain DAB Substrate kit (Cell Signaling #8059). Kidney sections stained for Collagen 3 were scanned using the Leica Aperio CS2 Scanscope at 20× and staining quantification was performed using Aperio Imagescope. Specifically for quantification, 27–31 separate cortical regions from each section were analyzed using the Positive Pixel Count v9 algorithm (5 animals per group), giving the ratio of total Collagen 3 positively stained pixels to the total number (stained and unstained) pixels in each region.

### 4.6. Muscle Fiber Typing and Measurements

EDL and Soleus muscles were frozen in isopentane cooled with liquid nitrogen and sectioned at eight microns. Fiber typing immunofluorescence was performed using MyHC subtype specific antibodies, Type I (clone BA-D5), Type 2a (clone SC-71) and Type 2b (clone BF-F3; Developmental Studies Hybridoma Bank, Iowa City, IA, USA). Briefly, sections were washed in PBS, blocked with anti-mouse Fab fragments (#715-007-003, Jackson ImmunoReseach, West Grove, PA, USA) and incubated with anti-MyHC antibodies for one hour at 37 °C. After washing in PBS, mouse IgG subtype-specific secondary antibodies (Jackson ImmunoResearch) were used for detection. Goat anti-mouse IgG2b Dylight 405 (#115-475-207) for Type I fibers, goat anti-mouse IgG1 Alexa 488 (#115-545-205) for Type 2a fibers and goat anti-mouse IgM Rhod Red X (#115-295-075) for Type 2b fibers. Rabbit anti-Laminin (#L9393, Sigma-Aldrich, St. Louis, MO, USA) was used to stain the muscle fiber sarcolemma and detected with anti-rabbit Alexa 647 (#A21245, ThermoFisher, Eugene, OR, USA). Slides were mounted with ProLong Gold (#P36934, ThermoFisher) and imaged with a Nikon A1R confocal microscope at 10× using the tiling feature to create a composite of the entire fiber. Muscle fibers stained for MyHC subtypes were measured using Fiji software. The cell counter feature was used to enumerate the different fiber subtypes. Blue-stained fibers are classified as Type 1, green fibers as Type 2A, red fibers as Type 2B and unstained fibers as Type 2X. Laminin staining (Far Red) was imaged as purple or white to outline the individual fiber membrane borders. Fiber sizes for each subtype were also measured and the values generated for minimum Feret diameter were used to evaluate fiber sizes.

### 4.7. Human Serum Treatment

For the experiments with uremic serum exposure, uremic serum was collected, prior to dialysis after the longest break (either on Monday or Tuesday), from a pool of stable in-center hemodialysis (HD) subjects. In total, 10 mL of uremic serum were drawn from ~100 HD subjects in their usual state of health (subjects were excluded if they had Hepatitis C, Hepatitis B or known or suspected acute infections). For the normal serum, 10 mL of normal serum was drawn from 50 kidney donors in their usual state of health before donation. Serum from HD subjects and kidney donors was pooled, filtered and aliquoted in small vials and frozen at −80 °C for cell experiments.

### 4.8. Cell Culture

C2C12 (CRL-1772, ATCC, Manassas, VA, USA) cells were used as a model of murine myoblasts/myotubes and cultured according to the manufacturer’s recommendations. Briefly, C2C12 cells were cultured in 10% fetal bovine serum (30-2020, ATCC) supplemented DMEM (30-2002, ATCC). For myotube formation, when confluent, myoblasts underwent differentiation by growth in DMEM containing 2.5% horse serum (16050130, Gibco) for five days.

### 4.9. Oxygen Consumption Rates (OCR) Measurement

C2C12 cells were plated and differentiated on Seahorse XF 24-well plates (100777-004, Agilent, Santa Clara, CA, USA). Cells were exposed to control or uremic serum for 24 h, and incubated in Seahorse XF DMEM medium (103334-100, Agilent), supplemented with 5 mM glucose (103577-100, Agilent) at 37 °C for 45 min without CO2 just before running the experiments. Seahorse XF Cell Mito Stress Test Kit (103015-100, Agilent) was used with the final respiratory modulators concentration of 1.5 µM for Oligomycin, 1 µM for FCCP and 0.5 µM for Rotenone/Antimycin A. OCR measurements were obtained using the Seahorse XFe24 Analyzer, and normalized to protein concentration (µg/µL). For the FAO experiments the Seahorse XF Palmitate Oxidation Stress Test Kit (102720-100, Agilent) was used following manufacturer’s recommendations. Similarly, cells were incubated for 24 h on substrate-limited medium supplemented with either control or uremic serum and then changed to FAO assay medium to incubate at 37 °C for 60 min. Prior to starting the assay, Palmitate-BSA substrate or BSA control was added to the cells. OCR was analyzed using Seahorse XFe96 Analyzer, and normalized to protein concentration (µg/µL).

### 4.10. Extracellular Lactate Measurement

C2C12 cells were exposed to serum in 12-well plates. After 24 h, cells were washed, and media replaced by fresh DMEM supplemented with 10% FBS. The cell culture supernatants were collected after 24 h and 48 h. Extracellular L-lactate concentration was measured using the EnzyChrom L-lactate Assay Kit (ECLC-100, BioAssay Systems) according to the manufacturer’s protocol. Results were normalized to total protein content (µg).

### 4.11. Extracellular H_2_O_2_ Production

C2C12 cells were seeded in 12-well plates with 2 × 10^5^ of cells per well and treated with 5% normal or uremic serum for 24 h. Then, cells were washed and incubated with DMEM media and 2.5 µM of PFBSF (10005983; Cayman, Ann Arbor, CA, USA). After 45 min, cells were washed and sorted on an LSR II Flow Cytometer (BD Biosciences, Franklin Lakes, NJ, USA). Data were analyzed using FlowJo 10.6 software.

### 4.12. Western Blot

Protein extraction, quantification and immunoblotting were performed as previously described [29]. Briefly, frozen gastrocnemius muscle was pulverized with mortar and pestle and homogenized in T-PER™ Tissue Protein Extraction Reagent (#78510, Thermofisher) with cOmplete™, Mini Protease Inhibitor Cocktail (#11836153001, Roche) and Halt™ Protease and Phosphatase Inhibitor Cocktail (#78442, Thermofisher). Primary antibodies used were anti-Glucose transporter 1 (#12939; Cell Signaling, Canvers, MA, USA), anti-Phosphoenolpyruvate carboxykinase (#8565; Cell Signaling), anti-Lactate dehydrogenase (#2012; Cell Signaling), anti-Monocarboxylate transporter 4 (sc-50329; SantaCruz), anti-Vinculin (#4650; Cell Signaling), anti-Translocase of the outer mitochondrial membrane complex subunit 20 (sc-17764; SantaCruz, Santa Cruz, CA, USA), anti-Glyceraldehyde-3-phosphate dehydrogenase (#2118; Cell Signaling), anti-Cytochrome c (#4280; Cell Signaling), anti-β-actin (A5441; Sigma) and anti-OXPHOS cocktail (MS601; Abcam, Cambridge, UK).

### 4.13. Statistical Analysis

All results were expressed as mean and standard error of the mean for normalized data or median and interquartile range for non-normalized data using GraphPad prism. Data were analyzed using two-tailed t tests for comparisons between two groups for normalized data and Mann–Whitney U test for non-normal data. A *p* value of <0.05 was considered statistically significant.

## Data Availability

Not applicable.

## Supplementary Materials

The following supporting information can be downloaded at: https://www.mdpi.com/article/10.3390/ijms232113515/s1, Figure S1: Renal histology of mice exposed to 0.2% adenine diet; Figure S2: Lactate production in C2C12 cells exposed to uremic serum.
