# Supplementary material for: Uremic Myopathy and Mitochondrial Dysfunction in Kidney Disease
Source: Int J Mol Sci. 2022 Nov 4;23(21):13515. doi: 10.3390/ijms232113515 (PMC9653774; doi:10.3390/ijms232113515)
Supplement: Supplementary file 1 [file ijms-23-13515-s001.zip › ijms-2008494-supplementary.pdf]

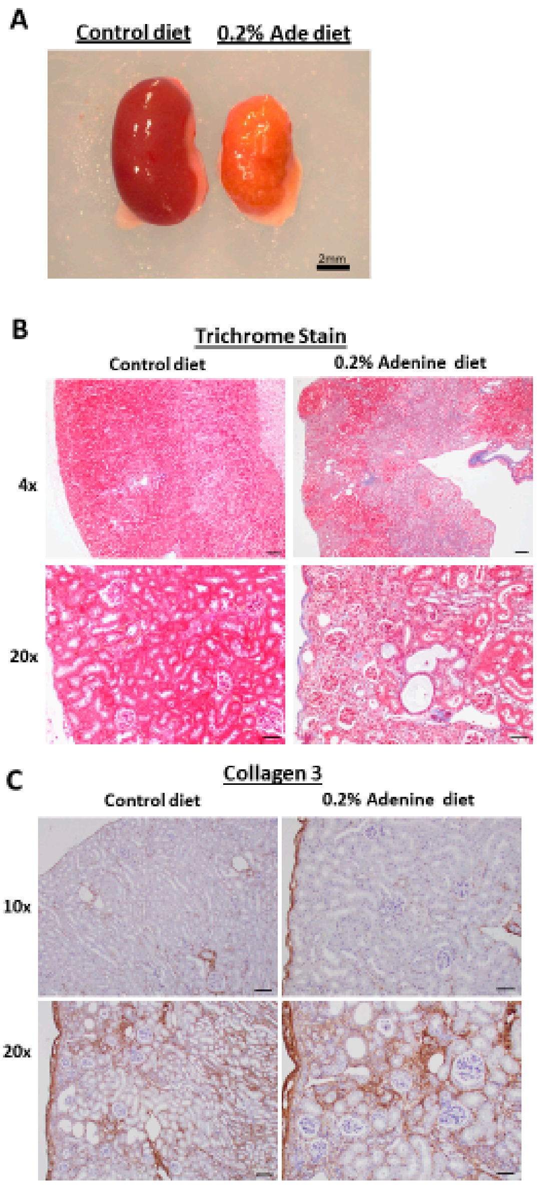

Supplementary Figure S1. Renal histology of mice exposed to 0.2% adenine diet. (A) Gross anatomy of representative kidneys from mice exposed to control and adenine diet. Scale bar 2 mm. Representative photographs of (B) Trichrome stain and (C) Collagen 3 immunostaining from sections of renal tissue of animals treated with control or adenine diet for 4 weeks. Scale bar 10X: 100 um, 20X 50 um for the Collagen images. 4x scale bar 200 um, 20X 50 um for the Trichrome images.

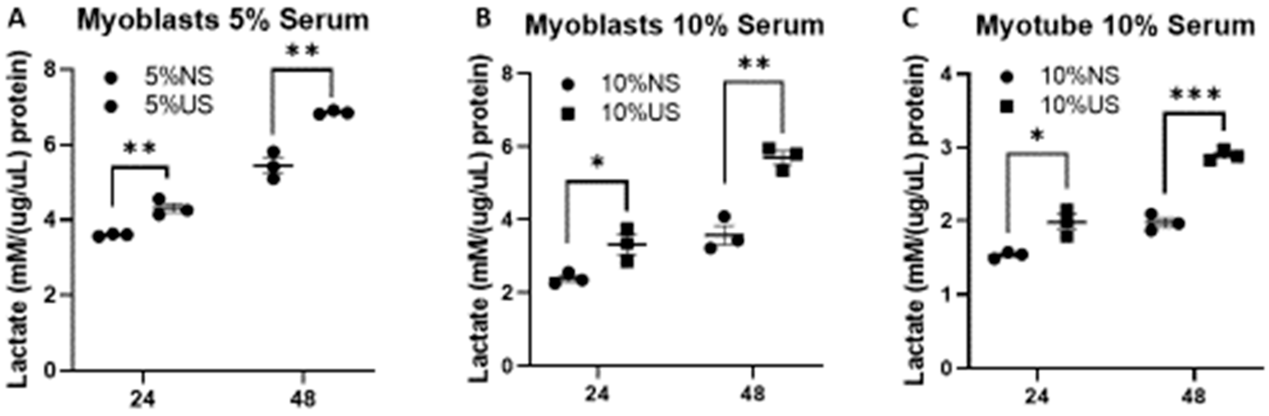

Supplementary Figure S2. Lactate production in C2C12 cells exposed to uremic serum. (A-B). Lactate production in myoblast exposed to 5%-10% uremic serum. (C) Lactate production in myotubes exposed to 10 % uremic serum for 24h. After 24h the serum is washed out and lactate production is measured 24h and 48h later in control media. Unpaired t test with Welch’s correction, error bars represent SEM. *p≤0.05, **p<0.01, ***p<0.001
